# Ecological footprint analysis of the phosphorus industry in China

**DOI:** 10.1007/s11356-022-20878-8

**Published:** 2022-05-27

**Authors:** Binlin Li, Danish, Salah Ud-Din Khan, Nils Haneklaus

**Affiliations:** 1 College of Economics and Management, Yunnan Agricultural University, Kunming, China; 2 School of Economics and Trade, Guangdong University of Foreign Studies, Guangzhou, 510006 China; 3 Sustainable Energy Technologies (SET) Center, College of Engineering, King Saud University, PO-Box 800, Riyadh, 11421 Saudi Arabia; 4 Institute of Chemical Technology, Freiberg University of Mining and Technology, 09599 Freiberg, Germany; 5 Td Lab Sustainable Mineral Resources, University for Continuing Education Krems, 3500 Krems an der Donau, Austria

**Keywords:** Ecological footprint, Phosphorus, China, Autoregressive distributive lag (ARDL)

## Abstract

Mitigating the effects of environmental deterioration requires a focus on not just CO_2_ emissions from energy consumption, but also environmental pollution from industry sectors. To reach this goal, recent studies have extended ecological footprint (EF) analysis to identify the ecological drivers of various key industry sectors. The role of the phosphorus (P) industry on the EF within the environmental Kuznets curve (EKC) framework for China is the emphasis of this study. Autoregressive distributive lag (ARDL) as well as the impulse response function and robustness analysis were used to consider a time from 1985 to 2018. The study verifies the EKC hypothesis for China in both the long and the short run, and indispensable determinants are proposed to be included to assure the model’s fitness and robustness when conducting EF analysis of industry sectors. Energy consumption–based carbon emissions have been verified as the dominant contributor to EF, but P use and urbanization have a significant lagged positive influence on EF in the short run. P exports, in particular, have been highlighted as a critical driver of the EF of China’s P industry. The conducted frequency domain causality test reinforced the above findings and demonstrated bidirectional causality at different frequencies. This work suggests that formulating plausible P export policies to alleviate the conflict between the output of China’s P industry and the environmental sustainability of this industry are necessary. In this context, “multidisciplinary, multidimensional, and practical solutions” are most desirable for sustainable P management.

## Introduction

The inclusive environmental degradation indicator “Ecological Footprint (EF)” contains three types of pollution (air, water, and soil), and is developed and used widely to offer insights into environmental degradation, demand for natural resources, and environmental pressure from economic activities (Arshad Ansari et al. 2020). Although EF has some limitations as recently acknowledged by the Global Footprint Network (GFN),^1^ it is still a widely used modelling tool that provides useful insides into the drivers of environmental degradation (Destek and Sinha 2020; Salemdeeb et al. 2021) helping countries to appraise their ecological resources (Solarin et al. 2019) derived from measurable, comprehensive, and easily understandable EF assessments (Ulucak and Lin 2017). EF analysis further contributes to policy-making, monitoring, and provides guidance on how to increase the sustainability of human activities (Arshad Ansari et al. 2020). Besides, recent analysis of multi-sectoral ecological sustainability issues facilitates the formulation of integrated strategies that incorporate policies, communities, research and innovation, and industrial action to address ongoing environmental degradation (Wang et al. 2021).

Quantifying the ecological and economic impacts from climate change, greenhouse gas (GHG) emissions, urbanization, and other drivers is challenging. Past studies have provided useful discussions on the nexus of natural resource use (mostly fossil fuel resources), emissions, and associated environmental degradation by using the hypothesis of the environmental Kuznets curve (EKC) in different countries and regions. Findings from these studies help contribute to the improvement of environmental quality (Ahmed et al. 2020; Danish et al. 2020b; Li and Haneklaus 2021). Several useful findings can be observed from recent studies. Firstly, these studies encouraged countries to increase their share of renewable energy solutions based on the findings related to the EF-energy nexus. The increasing shift from nonrenewable to renewable energy solutions has contributed to the improvement in ecological sustainability, and has thus fulfilled different important gaps and meanings in environmental economics (Destek and Sinha 2020). Secondly, EF analysis contributes to the availability and access to information through media pluralism and promotes greater awareness of environmental consciousness in the public. This enables people to jointly make efforts that contribute to sustainable environmental practices and standards (Langnel and Amegavi 2020). Thirdly, more and more recent empirical studies extended to different industry sectors that are contributing practical solutions that help the policy-making process within the EKC hypothesis. This again helps to create attention among policy makers and opinion leaders from academia and beyond. A recent example are the studies on the nexus of tourism and EF (Ozturk et al. 2016; Lee and Chen 2021; Sharif et al. 2020), hydropower and EF (Pata and Aydin, 2020), nuclear energy and EF (Danish et al. 2020a), and the natural gas industry–induced EKC (Li et al. 2021). Finally, in terms of variables selection, most of the recent EKC studies tend to focus on EF which includes diversified pollutants as the indicator of environmental degradation. Some indispensable variables such as economic growth, urbanization, trade, and resource use are usually included.

In this work, we focused on phosphorus (P) associated with finite phosphate rock (PR) resources that are paramount for P fertilizer (PF) production, and thus global food security as we know it today (Chen and Graedel, 2016; Li et al. 2019a). Moreover, due to P-related eutrophication and the associated impairment of fresh waterbodies, an extensive understanding of diversified ecological barriers in the P industry is vital to ensuring China’s ecological sustainability (Liu et al. 2016; Yan et al. 2021; Yuan et al. 2018). This work contributes to the existing literature by explicitly showing the key industry analysis of EF to the specific natural resources industry and investigate the nexus of “P-EF” within the EKC hypothesis in China. P utilization constitutes P use (PU) and P exports (PE) to display consequential effects on environmental degradation by incorporating indispensable determinants of economic growth, energy consumption–based carbon emissions, and urbanization. The autoregressive distributed lag (ARDL) model is used to indicate the long-run and short-run effects of P utilization on EF. Following Guan et al. (2020), the robustness analysis is shown by the long-run cointegration regression models of dynamic ordinary least squares (DOLS), fully modified ordinary least square (FMOLS), and canonical cointegrating regression (CCR), as well as the frequency domain causality test. At large, the study aims to focus on the P industry with the ultimate objective of fostering “*multidisciplinary, multidimensional and practical solutions*” to reduce the negative externality impact of the P industry in China and in extension elsewhere.

Existing literature on this topic is reviewed and analyzed in “Literature review.” The model specifications and econometric models are introduced in “Model specification and data.” “Empirical results” provides the study’s results and discussion. Conclusions, implications, and limitations are presented and discussed in “Conclusions, implications, and limitations.”

## Literature review

### Key industry sectors analysis of EF

Key industry sectors’ EF analysis at reginal or national level have gained increased attention from academics and policymakers as practical and quantifiable measurement tools for policy solutions to mitigate environmental degradation. Industry sectors analysis of EF is relevant as it provides meaningful insides to policy makers. Table 1 shows the summary of literature reviews on industry sectors conducted for this work that included EF analysis by testing the EKC hypothesis to indicate the impact of industry development on ecological degradation.

**Table 1:** Summary of ecological footprint (EF) analysis of different industry sectors within the EKC framework

Sectors	Authors	Countries/Region	Included variables
*Tourism-EF*	Liu et al. (2022)	Pakistan	Tourism, EF, foreign direct investment, Energy, trade
	Lee and Chen (2021)	123 countries	Tourism revenue, EF, GDP, country risk ratings
	Kongbuamai et al. (2020a)	ASEAN countries	Tourism, EF, GDP, energy consumption, natural resources
	Kongbuamai et al. (2020b)	Thailand	Tourism, EF, GDP, energy consumption, tradeopeness
	Katircioglu et al. (2018)	10 tourist countries	Tourism development, EF, GDP urbanization
*Finance-EF*	Abbasi et al. (2021)	Pakistan	Financial development, energy use, economic globalization index (EGI), GDP per capita, and technological innovation
	Destek and Sinha (2020)	11 newly industrialized countries	Financial development, EF, GDP, energy consumption
	Saud et al. (2020)	One belt one road initiative countries	Financial development, EF, globalization
	Baloch et al. (2019)	One belt one road initiative countries	EF, GDP, financial development, energy consumption, foreign direct investment, urbanization
*Agriculture-EF*	Udemba (2020)	India	EF, GDP, FDI agriculture, energy use, population
	Abdunnur (2020)	Indonesia	EF, fisheries production, agriculture production, urban development
	Pata (2021)	BRIC countries	EF, CO_2_, renewable energy, globalization, agriculture
*Biomass energy-EF*	Wang et al. (2020)	G7 countries	EF, biomass energy production
	Yasmeen et al. 2022	52 Belt & Road panel count	EF, biomass energy consumption,
*Nuclear energy-EF*	Danish et al. (2020)	China	EF, nuclear energy, CO_2_
*Electricity-EF*	Langnel and Amegavi (2020)	Ghana	EF, electricity consumption, GDP urbanization
*Social-political factors-EF*	Ahmed et al. (2020)	India	EF, human capitals energy consumption, GDP
	Khan et al. (2022)	18 Asian developing countries	EF; poverty; income inequality; GDP; forest area; inflation
	Charfeddine and Mrabet (2017)	15 MENA countries	EF, fertility rate, life expectancy, political institutional index
*hydropower energy-EF*	Pata and Aydin (2020)	Top six hydropower-consuming counties	EF, hydropower energy consumption, GDP

The association between tourism development and EF has gained attention in many studies and provides evidence for policy implications from tourism induced EKC analysis. Liu et al. (2022), for example, used the ARDL model to reveal the long-run cointegration association between tourism and EF in Pakistan during 1980–2017 within the theoretical EKC framework for sustainable tourism industry policy decision-making purposes. Sharif et al. (2020) provided a fresh insight into the investigation of the role of tourism development in China’s EF, suggesting that economic growth stimulates environmental degradation, and tourism exert a positive externality on EF in China. The variables of the two studies are very similar with a previous study by Ozturk et al. (2016) that investigated the effect of tourism development on EF in 144 countries. Key variables such as real income, energy use, trade, and urbanization were always tested in the tourism-EF nexus. Moreover, Lee and Chen (2021) revealed the association of tourism development and EF by incorporating factors of country risk ratings, and the political risk rating under the EKC framework, suggesting that ecological resources suffer negative externality from the increase of tourism revenues for the selected 123 countries.

Other important industry sectors such as finance, agriculture, biomass energy, natural gas energy, nuclear energy, human capital, social-political factors, and hydropower energy are also included in an active scientific discussion and are summarized in Table 1. Pata and Aydin (2020), for instance, explored the relationship between hydropower energy consumption, per capita of income, and EF under the EKC hypothesis for the top six hydropower-consuming countries in the world by using cointegration models covering the period of 1965–2016. The results indicated that there is no evidence that hydropower energy consumption and economic growth do not have a positive role in mitigating the EF. Danish et al. (2020a) analyzed the role of nuclear energy consumption on CO_2_ emissions under the EKC framework by using the ARDL model to show the long- and short-run dynamics covering the period from 1971 to 2018 in India. China’s natural gas industry–induced EKC hypothesis was further investigated by Li et al. (2021). The authors also discussed implications of addressing the PM2.5 emissions issue via developing a more comprehensive natural gas industry in China. Interestingly, a subject of extensive investigation is the analysis of the nexus between poverty, income inequality, and EF within the theoretical EKC framework. An example study is the work of Khan et al. (2022) that concluded that poverty has a positive association with EF and widening income inequality has a detrimental and harmful effect on environmental sustainability of 18 Asian developing countries during 2006–2017. More importantly, these kind of industry analysis using EF studies resulted in useful insights for policymakers and academia alike. Yasmeen et al. (2022), for instance, suggest practical and useful implications from the expanding deployment of advanced biomass production, foreign direct investment support, and stricter environmental-related policies that are proposed to secure China’s ecological sustainability.

### Environmental impact of phosphorus industry in China

China is the largest PR-producing country in the world (USGS 2021), and approximately 70% of the PR mined in China is used for PF production (Shang et al. 2021). PR mining, but particularly, later compound fertilizer production is energy-intensive. Ironically, humanity spends considerable energy on mining PR for its P content in some regions while applying the finite resource generously with the result of creating nutrient or P pollution in other areas that can even threaten food security and environmental sustainability from yet a different angle (Huang et al. 2019; Mekonnen and Hoekstra 2018). P is even considered as a long-term pollutant by some researchers (Li et al. 2020) since the current scale of global P flows from freshwater systems into the ocean has already transgressed the planetary boundary (Huang et al. 2019), as a result of modern agricultural operations and PR mining (Chen and Graedel 2016). In China, previous studies confirmed that large anthropogenic P inputs have caused widespread eutrophication of waterbodies, which compromise water quality and are detrimental for aquatic ecosystems (Bai et al. 2018; Huang et al. 2017; Jiang et al. 2019; Liu et al. 2012, 2021). Excessive fertilizer application, fertilizer production losses, and untreated sewage systems in China are pointed out to be potential causes for widespread eutrophication (Li et al. 2019a; Wu et al. 2020).

Recent studies further identify the environmental impact from P by using the environmental P footprint (Jiang et al. 2019; Li et al. 2019b; Shaw and Barnard, 2011). It is worth noting that these previous studies argue that China’s P footprint accounts for a large global proportion. Around 42% of total P exceedance footprint in the world was argued to come from China (Li et al. 2019b), and it is expected to keep increasing in the future. This increase largely results from an expected increase in the compounded consumption of meat and vegetables (Oita et al. 2020). Thus, it is urgent for China to take immediate actions to slow down the depletion of high-grade PR resources and reduce the overall P footprint (Jiang et al. 2019).

**Fig. 1:**
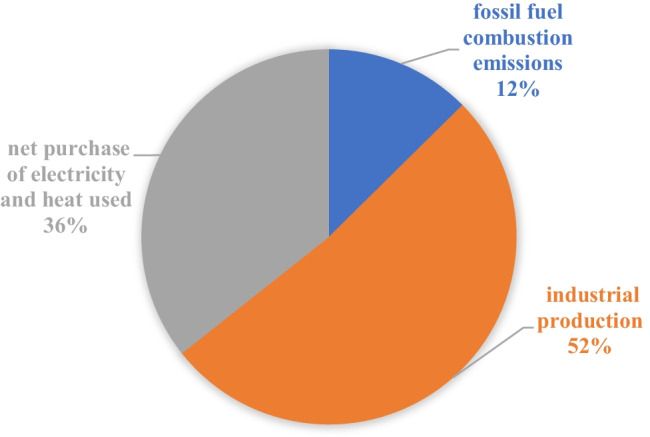
Sources of GHG emissions of PF production in China

More importantly, previous studies have provided evidence that confirm that P utilization is associated with ecological degradation. According to a report released from a Chinese P company about the GHG emission during P processing in 2019,^2^ total GHG emissions were estimated to be about 0.777 million tons of CO_2_ equivalent during PF production from the phosphoric acid and ancillary production units. Figure 1 provides an overview of the different fractions of the GHG emissions during PF production: (1) CO_2_ emissions from the industrial production, (2) emissions from the net purchase of electricity and heat used, and (3) fossil fuel combustion emissions. We can infer the large scale GHG emissions of the PF industry in China by considering the fact that the country has more than 100 PF companies. Chen et al. (2015) estimated GHG emissions from PF manufacturing in China. General GHG emission coefficients are estimated as 0.636 t of coal equivalent (tce)/t P_2_O_5_ produced. The estimated emissions are nearly two times higher than the emissions in developed countries. Moreover, Wu et al. (2020) also concluded that NPK compound fertilizer (15% P_2_O_5_) and diammonium phosphate (46% P_2_O_5_) production contribute even higher GHG emissions (CO_2_, CH_4_, and N_2_O). In addition, it can be argued that China’s emissions during P production are still significantly underestimated as only direct GHG emissions were considered in this estimate, thus leaving out indirect GHG emissions from land use change and additional GHG emissions from transportation and hauling.

P-related producers are further significant contributors to different pollutants, such as sulfuric acid emissions (Saeid and Chojnacka 2014); industrial sources of fluoride which easily flow into the food chain with an air–water interaction (Vallero 2020); waste streams containing radium and other waste byproducts from PR mining and PF production (Gad 2014); important sources for contamination of soils with heavy metals such as cadmium (McLaughlin et al. 2021), uranium (Haneklaus et al. 2017; Haneklaus 2021; Ye et al. 2019), and cobalt-containing waste (Lison 2007). Finally, high agricultural PF use rates are associated with environmental challenges for the environmental sources. P use not only has a considerable GHG footprint per cultivated area (Wu et al. 2020) but only 10–20% of the P fertilizer applied to soil is actually absorbed and utilized by crops (Hata et al. 2010). Most of the rest becomes immobilized in the inorganic and organic fractions of the soil and is thus unavailable to crops. It can then even flow into waterbodies leading to water pollution issues. The Global P loads to freshwater exceeds the assimilation capacity in 38% of the global land area, and about 30% of the anthropogenic P loads derive from China (Mekonnen and Hoekstra 2018).

To summarize, recent existing studies have extended the empirical investigation of the nexus of “Energy (or resource)-EF” to indicate the effect of different industry sectors on EF and reveal inconclusive outcomes and implications for practical policy decision-making. Besides, the key variables of real income, energy use, and urbanization (or trade) are always incorporated in the theoretical EKC framework to ensure the effectiveness of empirical findings. We could show that China’s P industry matters significantly for ecological degradation, and it is thus relevant to better understand the P-EF nexus within the EKC framework.

## Model specification and data

Given the aforementioned analysis, we provide a framework of the P-EF nexus that generalizes the key determinators of economic growth: energy consumption–based carbon emissions and urbanization incorporating P utilization (P use and P exports). Following Anwar et al. (2021) and Danish et al. (2020b), the estimation function is specified as follows:

1 $${EF}_{t}=f\left(GD,GD2, ECO, PU,PE,U\right)$$

*EF* denotes China’s environmental degradation indicator and can be considered as a critical indicator for designing policies to fight climate change (Salemdeeb et al. 2021). Additionally, economic development is represented as real income per capita (GD) (Danish et al. 2019). Incorporating its squared term (GD) aims to validate the theoretical EKC equations. ECO denotes energy consumption–based carbon emissions which were calculated using BP statistics from CO_2_ emissions derived from primary energy use. Urbanization (*U*) is incorporated to test the role of its inflows being an important determinant of environmental quality. The positive or negative effect of *U* on *EF* are controversial from unanimous findings by previous studies (Salman et al. 2022). Most importantly, we aimed to obtain the P industry as the key industry to be required to achieve sustainable resources management policy and encourage economic growth along with the reduction in problems of environmental degradation in China. The econometric model is as follows:

2 $${EF}_{t}={\beta }_{0}+{\beta }_{1}{\mathrm{RGDP}}_{t}+{\beta }_{2}{\mathrm{RGDP}}_{t}^{2}+{\beta }_{3}{\mathrm{ECO}}_{t}{+\beta }_{4}{PU}_{t}+{\beta }_{5}{PE}_{t}+{\beta }_{6}{\mathrm{LNUB}}_{t}+{\mu }_{t}$$

To get the direct elasticities of coefficients and to ease the process of estimating, the current study considered the natural log of the variables. The empirical logarithmic transformation (LN) form can be expressed as follows:

3 $${\mathrm{LNEF}}_{t}={\beta }_{0}+{\beta }_{1}\mathrm{LNGD}{P}_{t}+{\beta }_{2}\mathrm{LNGD}{P}_{t}^{2}+{\beta }_{3}\mathrm{LN}{{\mathrm{CO}}_{2}}_{t}{+\beta }_{4}{\mathrm{LNPU}}_{t}+{\beta }_{5}{\mathrm{LNPE}}_{t}+{\beta }_{6}{\mathrm{LNU}}_{t}+{\mu }_{t}$$

**Fig. 2:**
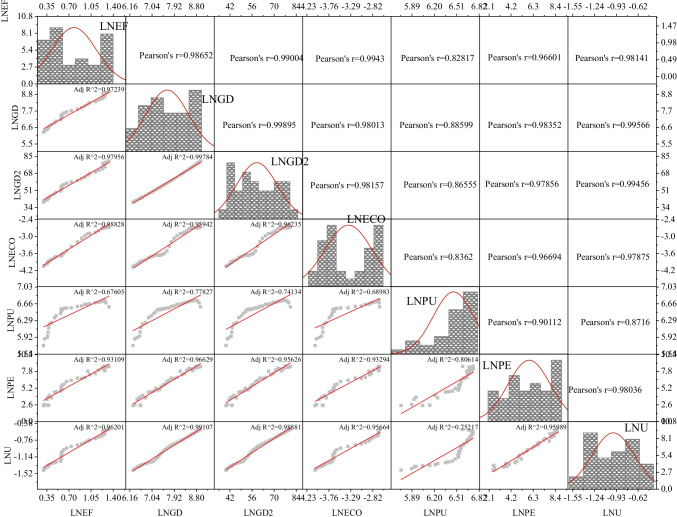
Scatter matrix graph of the logarithmic variables

The study uses an available annual dataset for China from 1985–2018. Table 2 presents detailed variables and sources. Table 3 provides the preliminary statistics. The scatter matrix graph presents the correlations among the logarithmic form of the variables, revealing that economic growth, ECO, PU, and PE, as well as *U* directly and indirectly cause environmental degradation in China (Fig. 2), suggesting that *EF* is more correlated with key factors of energy consumption and economic development, while real GDP is strongly linked to urbanization.

**Table 2:** Summary of detailed variables and source

Indicators	Abbrev	Unit	Source
Ecological footprint	*EF*	In global hectares	GFN^a^
Economic growth	GD	Constant 2010 US dollars	WDI^b^
Energy consumption-based carbon emissions	ECO	million t	BP statistics^c^
P use	PU	t	NBS^d^
P exports	PE	Thousand t of grand total P2O5	IFA^e^
Urbanization	U	%	WDI

*GFN*, Global Footprint Network; *NBS*, National Bureau of Statistics; *WDI*, World Development Indicators

^a^https://www.footprintnetwork.org/licenses/public-data-package-free/.

^b^https://databank.worldbank.org/reports.aspx?source=world-development-indicators.

^c^https://www.bp.com/content/dam/bp/business-sites/en/global/corporate/xlsx/energy-economics/statistical-review/bp-stats-review-2021-all-data.xlsx.

^d^https://www.qianzhan.com/.

^e^https://www.ifastat.org/.

**Table 3:** Preliminary statistics of logarithmic variables

	LNEF	LNGD	LNECO	LNPU	LNPE	LNU
Mean	0.8529	7.7626	8.4837	6.5428	6.2116	3.6725
Maximum	1.3122	8.9020	9.1376	6.7397	8.5885	4.0694
Minimum	0.3926	6.5683	7.7449	6.0376	2.5096	3.2661
Std. dev	0.3292	0.7399	0.5178	0.1881	1.8026	0.2697
Skewness	0.1293	-0.0315	0.0295	-1.1957	-0.4020	-0.1137
Kurtosis	1.4697	1.7511	1.3598	3.6075	2.1435	1.6144
Jarque–Bera	2.9104	1.8895	3.2551	7.3562	1.6675	2.3823
Obs	29	29	29	29	29	29

### Econometric methods

#### Unit root test

The reliability and effectiveness of the empirical results depend on the stationarity property of time-series data. Dickey-Fuller generalized least regression (DF-GLS) is proposed by Elliott et al. (1996) to improve the reliability of small-sample sizes. DF-GLS test do not allow considering structural break dates and we used the Zivot and Andrews (1992) structural break unit root test to validate the stationarity of the variables with structural break (Eqs. (4)–(6)).

4 $$\Delta {\xi }_{t}={\vartheta }_{1}+{\theta }_{2}t+\phi {\xi }_{t-1}+\psi D{\mathrm{um}}_{\mathrm{t}}+\sum_{i=0}^{r} {\omega }_{i}\Delta {\xi }_{t-i}+{\varepsilon }_{t}$$

5 $$\Delta {\xi }_{t}={\vartheta }_{1}+{\vartheta }_{2}t+\phi {\xi }_{t-1}+\delta D{\mathrm{trend}}_{t}+\sum_{i=0}^{r} {\omega }_{i}\Delta {\xi }_{t-i}+{\varepsilon }_{t}$$

6 $$\Delta {\xi }_{t}={\vartheta }_{1}+{\vartheta }_{2}t+\phi {\xi }_{t-1}+\psi D{\mathrm{um}}_{t}+\varphi D{\mathrm{trend}}_{t}+\sum_{i=0}^{r} {\omega }_{i}\Delta {\xi }_{t-i}+{\varepsilon }_{t}$$

$D{\mathrm{trend}}_{t}$ is the associated trend transition factor, whereas $D{um}_{t}$ is a dummy variable for the mean shift occurrences per each potential break-date (BD) (Eq. (7)).

7 $$\begin{array}{c}D{um}_{t}=\left\{\begin{array}{c}1\dots \dots . \, {\text{I}}{\text{f}} \, t>BD\\ 0\dots \dots .. \, {\text{o}}{\mathrm{t}}{\text{h}}{\mathrm{e}}{\text{r}}{\mathrm{w}}{\text{i}}{\mathrm{s}}{\text{e}} \, \end{array}\right.\\ D{\mathrm{trend}}_{t}=\left\{\begin{array}{c}t-BD\dots . \, {\text{I}}{\mathrm{f}} \, t>BD\\ 0\dots \dots \dots \dots \, {\text{o}}{\mathrm{t}}{\text{h}}{\mathrm{e}}{\text{r}}{\mathrm{w}}{\text{i}}{\mathrm{s}}{\text{e}}\end{array}\right.\end{array}$$

## ARDL model

To investigate the long-run cointegration relationship between the variables, the literature provides several econometric approaches such as FMOLS introduced by Phillips and Hansen (1990) and DOLS introduced by Stock and Watson (1989) for cointegration analysis. In more recent studies, ARDL is widely used because of its advantage of allowing a small-sized sample to indicate both long- and short-run dynamics. The method can further accommodate serial correlation and endogeneity among the variables by using reasonable lag selection and robustness estimates, while not allowing all variables to be integrated with the same order in comparison with Johansen’s cointegration technique. The empirical equation of the ARDL for this study is shown in Eq. (8):

8 $$\begin{array}{c}\Delta {\mathrm{LNEF}}_{t}={\sigma }_{0}+\sum_{k=1}^{P} {\varnothing }_{1k}\Delta \mathrm{LN}E{F}_{t-k}+\sum_{k=0}^{P} {\varnothing }_{2k}\mathrm{\Delta LN}{\mathrm{GDP}}_{\mathrm{t}-\mathrm{k}}+\sum_{k=0}^{P} {\varnothing }_{3k}\Delta \mathrm{LN}{{\mathrm{GDP}}^{2}}_{t-k}+\sum_{k=0}^{P} {\varnothing }_{4k}\mathrm{\Delta LN}{{\mathrm{CO}}_{2}}_{t-k}\\ +\sum_{k=0}^{P} {\varnothing }_{5k}\Delta {\mathrm{LNPU}}_{t-k}+\sum_{k=0}^{P} {\varnothing }_{6k}\Delta {\mathrm{lLNPE}}_{t-k}+\sum_{k=0}^{P} {\varnothing }_{7k}\mathrm{\Delta LN}{\mathrm{U}}_{t-k}+{\beta }_{1}{\mathrm{LNEF}}_{t-1}+{\beta }_{2}{\mathrm{LNGDP}}_{\mathrm{t}-1}\\ +{\beta }_{3}\mathrm{LN}{{\mathrm{GDP}}^{2}}_{\mathrm{t}-1}+{\beta }_{4}\mathrm{LN}{{\mathrm{CO}}_{2}}_{t-1}+{\beta }_{5}{\mathrm{LNPU}}_{t-1}+{\beta }_{6}{\mathrm{LNPE}}_{t-1}+{\beta }_{7}{\mathrm{LNU}}_{t-1}+{\gamma \mathrm{ECMt}-1 +\varepsilon }_{1t}\end{array}$$

where $\Delta$ represents the first difference operator and ${\varepsilon }_{1t}$ the random error terms. The short-run coefficients of the model are denoted by ${\varnothing }_{1}$–${\varnothing }_{7}$. ${\beta }_{1}$–${\beta }_{7}$ are the long-run coefficients. ${\mathrm{ECM}}_{t-1}$ denotes the error correction term and *γ* is the adjustment coefficient. The *F*-test is employed for bound tests to examine the combined significance of the lagged levels in the equations. The null hypothesis (${H}_{0}$) is given by Eq. (9):

9 $${{H}_{0}=\beta }_{1}={\beta }_{2}={\beta }_{3}={\beta }_{4}{=\beta }_{5}{=\beta }_{6}{=\beta }_{7}=0$$

Thus, the short-run association can be expressed as Eq. (10):

10 $$\begin{array}{c}\mathrm{\Delta LNE}{\mathrm{F}}_{\mathrm{t}}={\sigma }_{0}+\sum_{k=1}^{P} {\varnothing }_{1k}\Delta {\mathrm{LNEF}}_{t-k}+\sum_{k=0}^{P} {\varnothing }_{2k}\mathrm{\Delta LN}{\mathrm{GDP}}_{t-k}+\sum_{k=0}^{P} {\varnothing }_{3k}\Delta \mathrm{LN}{{\mathrm{GDP}}^{2}}_{t-k}+\\ \begin{array}{c}\\ \sum_{k=0}^{P} {\varnothing }_{4k}\Delta LN{{CO}_{2}}_{t-k}+\sum_{k=0}^{P} {\varnothing }_{5k}\Delta {\mathrm{LNPU}}_{t-k}+\sum_{k=0}^{P} {\varnothing }_{6k}\Delta {\mathrm{LNPE}}_{t-k}+\sum_{k=0}^{P} {\varnothing }_{7k}\Delta LN{\mathrm{U}}_{t-k}\\ +{\gamma \mathrm{ECMt}-1 +\varepsilon }_{1t}\end{array}\\ \end{array}$$

## FMOLS, CCR, and DOLS

FMOLS estimates include a completely asymptotic ordinary mix which permits for standard Wald testing by utilizing the asymptotic inference of Chi-square and employ the precursory assessment of the symmetric and of the residuals of the long-run covariance matrices. The estimates are shown in Eq. (11):

11 $$\widehat{\theta }=\left[\begin{array}{c}\beta \\ {\widehat{\gamma }}_{1}\end{array}\right]={\left(\sum_{t=2}^{T} {Z}_{t}{Z}_{t}^{\mathrm{^{\prime}}}\right)}^{-1}\left(\sum_{t=2}^{T} {Z}_{t}{\vartheta }_{t}^{+}-T\left[\begin{array}{c}{{\varvec{X}}}_{12}^{+}\\ 0\end{array}\right]\right)$$

where dependent variable $\vartheta$ followed by regressors ${\varvec{X}}$. $\widehat{\Omega }$ and $\widehat{\lambda }$ represent long-run covariance matrices using the residuals ${\widehat{u}}_{t}$ that can be shown as follows:

12 $${\vartheta }_{t}^{+}={\vartheta }_{t}-{\widehat{\omega }}_{12}{\widehat{\Omega }}_{22}^{-1}{\widehat{u}}_{2}$$

13 $${\widehat{\lambda }}_{12}^{+}={\widehat{\lambda }}_{12}-{\widehat{\omega }}_{12}{\widehat{\Omega }}_{22}^{-1}{\lambda }_{22}$$

14 $${Z}_{t}=\left({X}_{t}^{^{\prime}},{D}_{t}^{^{\prime}}\right)$$

Scalar estimator ${\widehat{\omega }}_{1.2}$ can be indicated as follows:

15 $${\widehat{\omega }}_{1.2}={\widehat{\omega }}_{11}-{\widehat{\omega }}_{12}{\widehat{\Omega }}_{22}^{-1}{\widehat{\omega }}_{21}$$

Unlike FMOLS, CCR also requires a consistent estimate of the contemporaneous covariance matrix $\widehat{\Sigma }$. CCR estimates can be given by the following:

16 $$\left[\begin{array}{c}\beta \\ {\widehat{\gamma }}_{1}\end{array}\right]={\left(\sum_{t=1}^{T} {Z}_{t}^{*}{Z}_{t}^{{*}^{\mathrm{^{\prime}}}}\right)}^{-1}\sum_{t=1}^{T} {Z}_{t}^{*}{\vartheta }_{t}^{*}$$

The DOLS estimates also have the same asymptotic distribution with FMOLS and CCR estimates, and remedies for a few of the bias result from the endogeneity issue (Månsson et al. 2018). DOLS technique involves augmenting the cointegrating regression with lags and leads of $\Delta {X}_{t}$ so that the resulting cointegrating equation can be shown as follows:

17 $${\vartheta }_{t}^{*}={\vartheta }_{t}-{\left({\widehat{\Sigma }}^{-1}{\widehat{\Lambda }}_{2}\stackrel{\sim }{\beta }+\left[\begin{array}{c}0\\ {\widehat{\Omega }}_{22}^{-1}{\widehat{\omega }}_{21}\end{array}\right]\right)}^{\mathrm{^{\prime}}}{\widehat{u}}_{t}$$

It is worth noting that robustness analysis is necessary to test the reliability of FMLOS, CCR, and DOLS estimates, efficiency of parameters, and nuisance free parameters for the estimated chi-square tests.

## Frequency domain causality test (FDCt)

This analysis is motived by Abbasi et al. (2021), and frequency domain causality test (FDCt) that was proposed by Breitung and Candelon (2006) based on the previous method of Hosoya (1991) are employed in this section to capture the causality effects of economic growth, energy consumption–based carbon emissions, P utilization, and urbanization on *EF* at different frequencies in China for robustness analysis purposes. The FDCt can display nonlinear causal effects to effectively eliminate seasonality changes in the smaller sized time sequences and enables the classification the long-, medium-, and short-term causalities across determinants at different frequencies (He et al. 2021). This FDCt with Hosoya (1991) method can be demonstrated as the following econometric process:

A two-dimensional time-series vector $\left|{x}_{t},{y}_{t}\right|$ with ${R}_{t}$ can be obtained from a VAR analysis framework in Eqs. (18)–(19):

18 $$\theta \left(L\right){R}_{t}={\upsilon }_{t}$$

19 $$\Theta \left(L\right)=1-{\Theta }_{1}L-\dots -{\Theta }_{p}{L}^{p}$$

where ${\upsilon }_{t}$ denotes the error term vector and the moving average (MA) simulation was calculated using Eq. (20) after verifying the stationary property of time sequences:

20 $${R}_{t}=\Theta \left(L\right){\upsilon }_{t}=\left[\begin{array}{c}\begin{array}{cc}{\Omega }_{11}(L)& {\Omega }_{12}(L)\end{array}\\ \begin{array}{cc}{\Omega }_{21}(L)& \&{\Omega }_{22}(L)\end{array}\end{array}\right]\left[\begin{array}{c}{\mu }_{1t}\\ {\mu }_{2t}\end{array}\right]$$

where ${\Omega }_{11}$ and $\mu$ represent the coefficient pattern and white noise, respectively. Afterwards, the spectral density of ${x}_{t}$ based on the representation is given by the following:

21 $${f}_{x}\left(\omega \right)=\frac{1}{2\pi }\left\{\left|{\Omega }_{11}\left({e}^{-i\omega }\right)\right|2+\left|{\Omega }_{12}\left({e}^{-i\omega }\right)\right|2\right\}$$

According to Hosoya (1991), an estimate of the causality that incorporates frequency ($\omega$) can be determined using the following:

22 $${\rm H}_{y\to x}\left(\omega \right)=\mathrm{log}\left[\frac{2\pi {f}_{x}\left(\omega \right)}{\left|{\Omega }_{11}\left({e}^{-i\omega }\right)\right|2}\right]=\mathrm{log}\left[\frac{\left|{\Omega }_{12}\left({e}^{-i\omega }\right)\right|2}{\left|{\Omega }_{11}\left({e}^{-\omega }\right)\right|2}\right]$$

where if $\left|{\Omega }_{12}\left({e}^{-i\omega }\right)\right|\ne 0$, $y$ is validated to affect $x$ at frequency(ω). If ${R}_{t}=\left|{x}_{t},{y}_{t}\right|$ is verified to be cointegrated, $\theta (L){R}_{t}={\upsilon }_{t}$ can be defined as Eq. (23) and can be simplified as shown in Eq. (24):

23 $$\Delta {R}_{t}=\left({\theta }_{1}-I\right){R}_{t-1}+{\theta }_{2}{R}_{t-2}+\dots +{\theta }_{p}{R}_{t-p}+{\upsilon }_{t}=\overline{\theta }\left(L\right){R}_{t-1}+{\upsilon }_{t}$$

24 $$\Delta {R}_{t}=\varpi \left(L\right){\varepsilon }_{t}=\overline{\vartheta }\left(L\right){\eta }_{t}$$

where $\mathrm{\varpi }(L)=\mathrm{\varpi }(L){G}^{-1},{\upsilon }_{t}={G}_{\upsilon t}$ and *G re*present a triangle matrix correlated with $E\left({\mu }_{t}{\mu }_{{t}^{\mathrm{^{\prime}}}}\right)=I$*.* Then, Hosoya (1991) proposed the causality association of the stationary time sequences as shown in Eq. (25):

25 $${H}_{y\to x}\left(\omega \right)=\mathrm{log}\left[1+\frac{\left|{\Omega }_{12}\left({e}^{i\omega }\right)\right|2}{\left|{\Omega }_{11}\left({e}^{-i\omega }\right)\right|2}\right]$$

Thus, the ${H}_{y\to x}(\omega )=0$ indicate that the null hypothesis of $y$ does not forecast $x$ at different frequencies.

The FDCt at frequency ($\omega$) between ${x}_{t}$ and ${y}_{t}$ was further introduced by Breitung and Candelon (2006) in the following form:

26 $${x}_{t}={\alpha }_{1}{x}_{t-1}+\dots +{\alpha }_{q}{x}_{t-q}+{\beta }_{1}{y}_{t-1}\dots +{\beta }_{q}{y}_{t-q}+{\upsilon }_{1t}$$

## Empirical results

### Unit root tests and lag lengths selection

Table 4 presents variables for the first difference of the variables test that were checked for stationarity using unit root test techniques. The literature provides multiple tests for stationarity; however, in this study, while the empirical study employs a relatively small-sized dataset sample, the Dickey-Fuller generalized least regression (DF-GLS) test was used to identify the stationary features of all variables with the natural logarithm as better performance for testing the stationary of small sample–sized datasets. The results of the DF-GLS and Zivot-Andrews’ (1992) structural break tests on the first difference of the variables support the idea that all data series are stationary. Thus, all the variable series were verified to be integrated of order I (1). We also provided more reliable results from Zivot-Andrews (ZA) structural break tests to detect the break point (*T*_*y*_). We could only observe that LNU has a break year in 1996, which was argued as transitioning from a steady phase of ascension (1979–1995) to a rather fast promotion phase (1996–2010) (Chen et al. 2013). The dependent variable of LNEF, regressors of LNGD, LNECO, and LNPE have break points in 2001 and 2002, which can be explained by the related environmental laws and regulations that started to be implement since 2000 in China. LNPU has a break year in 2004 that might be a result of the attention and resulting industrial best practice solutions from agriculture on P efficiency and P pollution in China.

**Table 4:** Unit root test results by DF-GLS and ZA structural break test

	DF-GLS		ZA structural break			
	Level	1^st^	Level		1^st^	
	*t*-stat	*t*-stat	*t*-stat	*T*_*y*_	*t*-stat	*T*_*y*_
LNEF	− 0.2089	− 2.999a	− 4.9576a	1996	− 3.6838a	2001
LNGDP	− 0.3565	− 2.551b	− 3.5489	2006	− 4.9077b	2011
LNGDP^2^	− 0.4260	− 2.2963b	− 4.0537	2002	− 4.5791b	2011
LNCO_2_	− 0.5638	− 2.1794b	− 5.4936	2003	− 3.9920a	2002
LNPU	0.7215	− 4.1137a	− 1.49566	2013	− 5.7889b	2004
LNPE	− 0.9094	− 7.3120a	− 4.9694a	2013	− 5.3252b	2002
LNU	− 0.186	− 2.0121b	− 3.0926	2013	− 7.8198a	1996

a, b, c represent the significance of 1%, 5%, and 10%, respectively

Lag lengths selection are crucial for cointegrating models. Table 5 shows the result for lag order selection, and five criteria: likelihood ratio (LR), final prediction error (FPF), Akaike information criterion (AIC), Schwarz information criterion (SC), and Hannan-Quinn information criterion (HQ) that supports the maximum lag length of 3 for conducting cointegration in the next steps

**Table 5:** The result of lag selection technique

Lag	LogL	LR	FPE	AIC	SC	HQ
0	171.8825	NA	5.66e-14	− 10.6376	− 10.3139	− 10.5320
1	550.7862	562.2442	3.51e-23	− 31.9217	− 29.3313	− 31.0773
2	630.5712	82.3587	8.23e-24	− 33.9078	− 29.0508	− 32.3245
3	750.5512	69.6658*	5.41e-25*	− 38.4872*	− 31.3635*	− 36.1650*

### Results from ARDL

#### Bound tests for cointegration and diagnostic test

The results of bound tests for cointegration for the selected ARDL model (1, 3, 3, 1, 2, 2, 3) have been reported in Table 6. The results indicate that *F*-statistic values are higher than the critical value of the upper bound, and statistically significant at the 1% level. For the diagnostic test (Table 7), heteroscedasticity, Ramsey Regression Equation Specification Error Test (RESET), J-B normality, and serial correlation were conducted. The outcome supports that serial correlation and heteroskedasticity does not exist in the selected model and rejects the hypothesis of normal distribution, suggesting that the selected ARDL model is generally sufficient to provide the magnitude of elasticity required for the analysis conducted here. In addition, the RESET is used to testify the accuracy and stability of the model.

**Table 6:** Bounds test for cointegration results

	Model		*F*-stat	Conclusion
${EF}_{t}=f(GD,GD2, \mathrm{ECO}, PU,PE,U)$	ARDL (1, 3, 3, 1, 2, 2, 3)		9.2072	Cointegration
Critical values	1%	2.50%	5%	10%
Lower bounds I(0)	2.88	2.55	2.27	1.99
Upper bounds I(1)	3.99	3.61	3.28	2.94

**Table 7:** Results of the diagnostic tests

Diagnostic test	*F*-stat	*P*-value	Result
Breusch-Godfrey LM	14.5885	0.1999	√
Breusch-Pagan-Godfrey	1.3995	0.3098	√
J-B test	0.7660	0.6818	√
Ramsey RESET	1.6122	0.2399	√

**Fig. 3:**
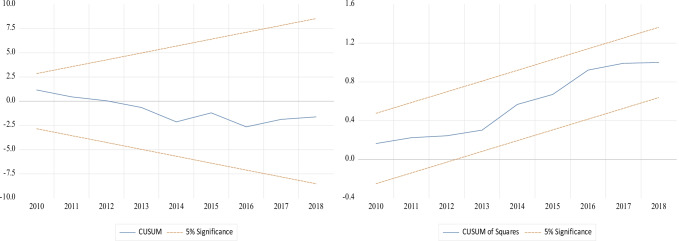
Graphs of the stability test from CUSUM/CUSUMQ stability test

The cumulative sum of recursive residuals (CUSUM) and cumulative sum of squares of recursive residuals (CUSUM of squares) are employed to test the stability of the models. Figure 3 displays the graphs of the CUSUM and cumulative sum of recursive residual square (CUSUMQ) that are within the 5% critical bounds within the 95% confidence interval, implying parameter consistency and stability of the selected ARDL (1, 3, 3, 1, 2, 2, 3) model. The data suggests that the obtained results can be efficiently used to estimate the long-run and short-run association between the EF nexus and other variables by using the confirmed goodness and fitness ARDL model.

#### Long-and short-run estimation of the ARDL

Table 8 presents the results of ARDL estimate of long-run and short-run coefficients to indicate the nexus of EF and per capita GDP, energy consumption–based carbon emissions, PU, PE, *U* for China in 1985–2018. We can observe that the statistical significance of the *CointEq(− 1)* suggests convergence of the dynamics from the short-run to the long-run equilibrium that significantly validates the stability of the ARDL model, implying that deviation from the short run towards the long run are corrected back by as high as 78.62% and will take time to reach the long-run equilibrium.

**Table 8:** Results for long-run and short-run relationship

Variable	Coefficient	Std. Error	t-Statistic	Prob
Long run				
LNGD	7.1802	0.7718	9.3027	0.0000
LNGD2	− 0.3387	0.0380	− 8.9205	0.0000
LNECO	0.2264	0.0679	3.3365	0.0087
LNPU	− 1.7254	0.1853	− 9.3122	0.0000
LNPE	0.0435	0.0167	2.6027	0.0286
LNU	− 3.6657	0.3045	− 12.0389	0.0000
C	− 25.8270	2.8378	− 9.1010	0.0000
Short run				
DLN (GD)	2.8698	0.8636	3.3230	0.0089
DLN (GD(− 1))	− 1.0001	1.1372	− 0.8794	0.4020
DLN (GD(− 2))	− 6.1223	0.9583	− 6.3891	0.0001
DLN(GD2)	− 0.1260	0.0599	− 2.1054	0.0645
DLNGD2 (− 1)	0.0213	0.0778	0.2736	0.7905
DLNGD2 (− 2)	0.3212	0.0616	5.2146	0.0006
DLNECO	0.5081	0.0436	11.6632	0.0000
DLNPU	− 0.0747	0.0592	− 1.2609	0.2390
DLNPU (− 1)	0.8905	0.1220	7.3017	0.0000
DLNPE	− 0.0043	0.0045	− 0.9654	0.3596
DLNPE (− 1)	− 0.0317	0.0050	− 6.3379	0.0001
DLNU	− 3.2860	0.3267	− 10.0583	0.0000
DLNU (− 1)	0.5848	0.2895	2.0202	0.0741
DLNU (− 2)	0.9752	0.2550	3.8247	0.0041
*CointEq (− 1)*	− 0.7862	0.0687	− 11.4432	0.0000

**Fig. 4:**
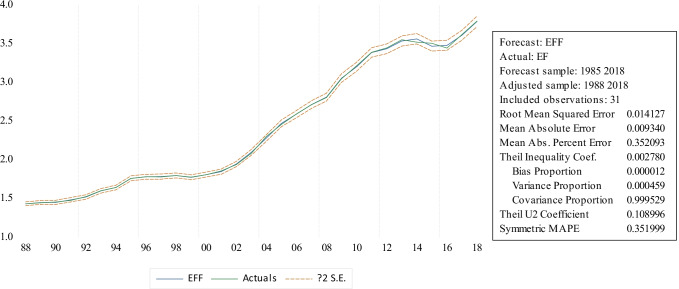
Actual value of EF and static forecasted *EF* (EFF) during 1985–2018 via ARDL estimation

In the case of economic growth, the results of the ARDL indicate that the long-run coefficient between economic growth (real income per capita) and *EF* are positive and significant (Fig. 4), while the square term of real income per capita has a statistically significant and negative relationship with *EF* both in the long and short run. Our findings are consistent with the previous EKC hypothesis argument by Danish et al. (2020b) and Lee and Chen (2021) for China and confirm one more time the existence of the EKC when incorporated with the P industry in the long- and short-run scenario for China. Specifically, if the per capita income rises by 1%, this will cause the EF to increase by 7.18% which has a smaller impact than the previous conclusion from Danish et al. (2020b) (11.841%). This result highlights that economic growth is the most important driver for ecological degradation in China over the sample period, and in line with the EKC hypothesis argument that as a country’s economic growth increases, it tends to shift towards more stringent environmental policies. Thus, we can continue to use the theoretical EKC framework to indicate the elasticity of other determinants on EF for China.

Regarding the energy consumption sector, the findings reveal a long-run 1% increase of ECO stimulates a 0.226% increase of EF and a higher short-run contribution on ecological degradation of 0.51%, implying that long-run environmental degradation is highly correlated with energy consumption, and that short-run effects stimulate much more environmental deterioration in China. It was further found that changes in the long-run urbanization have a negative and statistically significant influence on *EF*.

In terms of *EF* analysis of China’s P industry, the ARDL results for the long-run relationship between *EF* and PU are statistically significant and negative, and if it rises by 1% this will cause the *EF* to decrease by 1.725% in the long run, and in the short-run estimation, we can observe that the first lag of PU has a positive effect on ecological degradation changes. If PU rises by 1%, this will cause the EF to increase by 0.89%, suggesting that the response to the contemporary change of *EF* is embodied in the previous year’s P use. The long-run negative role of PU on environmental degradation is shown by the relationship of natural resources use and EF in China as explained by Danish et al. (2020b). The effect of PU on environmental degradation might be explained by (1) a series of policies to promote sustainable P management and increase PU efficiency, and (2) PU that is always connected with the development of the agricultural economy. Our findings indicate that although rapid development of the agriculture economy leads to increasing P consumption, the major contributor to environmental degradation is found in the industrial sectors. With regards to the nexus of PE and EF, our findings indicate that PE is an important source to the ecological degradation for China in the long run. Specifically, in the long run, a 1% increase in PE will lead to a 0.044% increase of EF. This result verifies the specification that the large-scale increase of PE possibly caused by expansion of production capacity significantly exacerbates negative externality in all the supply chain of the P industry and leads to a significant increase of *EF*. Besides, the rise of PE indicates the depletion of higher grade PR resources that results in increased demand of input resources such as energy and water which all have an impact on environment degradation. Furthermore, it is evident that Chinese P companies that rely more on exports are generating higher environmental costs.

In the case of urbanization, a 1% increase of the long-run change in the urbanization rate leads to a 3.666% decrease of the *EF* for ARDL results, while a 1% increase of the contemporaneous change in the short-term urbanization rate leads to a 3.286% decrease of the EF. More importantly, our findings reveal that the 1^st^ and 2^nd^ lag of urbanization significantly contributed to the ecological degradation with 0.58% and 0.98%, respectively, suggesting a short-run positive impact on EF in the previous 1 and 2 years. The result of the long-run coefficient is again consistent with the results from Danish et al. (2020b). Our results support the argument that urbanization shows productive land resources utilization more precisely, and thus helps controlling natural resources depletion (Danish et al. 2020b). The positive externalities from urbanization might derive from the return to economies of scale and public services supply, i.e., waste management policy and more environment-friendly infrastructure (Danish et al. 2019). Besides, urban citizens tend to have a better awareness and tendency to take initiatives to protect the environment (Danish et al. 2020b). Most importantly, this urbanization is a key driver for using more and more advanced technologies (i.e., the deployment of 5G infrastructure) to create smart green cities in China. A good example may be the fast transformation from fossil-fueled vehicles to new energy vehicles (electric, fuel cell, or plug-in hybrid) in Chinese cities and that by 2035 half of all new vehicles sold in China will indeed be new energy vehicles^3^.

**Fig. 5:**
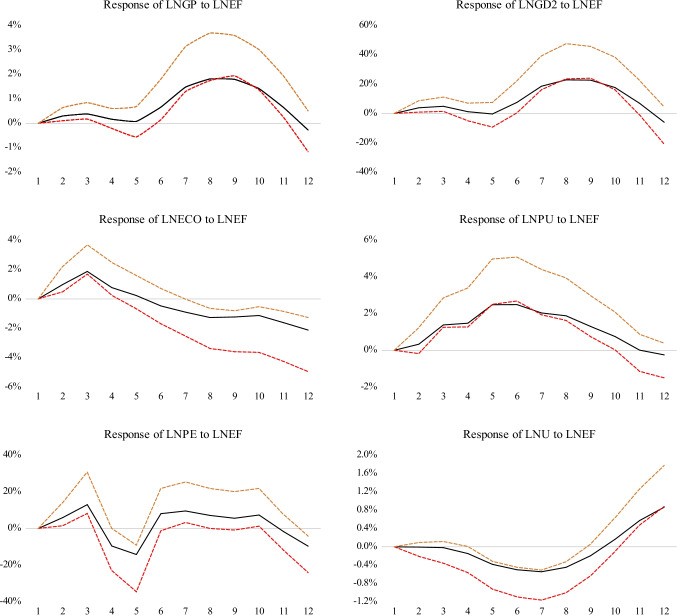
Graphs of the IRF test results

The presented analysis further provides the static forecasting performance of ARDL. Mean absolute error (MAE), root mean square error (RMSE), symmetric mean absolute percent error (SMAPE), and Theil inequality coefficients (TIC) are used to indicate the accuracy of the static forecast results. Figure 5 confirms that the selected ARDL model introduced smaller values of MAE, RMSE, and MAPE, and TIC that are closer to 0, implying that above estimates show excellent static forecasting performances in assessing the elasticity of *EF* for GD, ECO, PU, PE, and *U*. This again indicates that the ARDL model has excellent fitness and goodness to forecast the *EF* value during the regarded period from 1985 to 2018.

### Impulse response function (IRF) analysis

In this section, we used impulse response function (IRF) analysis to further indicate dynamic impacts of GD, ECO, PU, PE, *U*, on *EF* over a 12-year period. The 95% confidence interval bands are computed by the newly developed bootstrapping approach with 999 repetitions.

Figure 5 shows the results of the IRF graphs for the nexus of *EF* and other variables. The response of *EF*s to per capita income and its square term are positive starting from the 5th year and the intensity of the response increased and peaked during the 7–8th year. After that, it declined significantly showing the characteristic EKC pattern that validates our result from the ARDL model. When a standard deviation impact of ECO and *EF* is provided, a positive response to ECO in both the short and long run can be observed. The response of the short-run effect has a much stronger effect on *EF* than long-run effects do, which is consistent with the result from the ARDL, reconfirming that the response of *EF* would obtain the equilibrium drift in both short- and long-time periods, but short-run impacts show stronger effects on EF. In the cases of the EF-PU and EF-PE nexus, the response of EF will be significantly stronger towards PE than PU, implying that P exports have stronger effects on *EF* than PU. It further shows that *EF* is more sensitive to the changes of PE in short periods (1–3 year period) and in long-run periods (6–10-year period). Besides, a positive one standard deviation impact of urbanization exerts a significant negative impact on *EF* from the third year and a positive effect starting from the 9th year. This means that the reaction of *EF* to urbanization differs greatly during different stages of urbanization in China.

### Robustness analysis

To ensure the robustness of the ARDL results, we used the long-run cointegration methods of FMOLS, CCR, and DOLS for comparing the long-run relationship with the ARDL findings. The output of the estimates is shown in Table 9. All the statistical results are consistent with the signs of their coefficients but show different effects of magnitude for the results of the ARDL. This means that the outcomes of elasticity of EF for GD, ECO, PU, PE, and *U* from the ARDL are useful and suitable to indicate the impact on environmental degradation in positive or negative ways in the long run. More importantly, our result supports the idea that DOLS have better performance when applied to the robustness check in this study.

**Table 9:** The long-run relationship results from FMOS, CCR, and DOLS

	FMOLS		CCR		DOLS	
Variable	Coefficient	Prob	Coefficient	Prob	Coefficient	Prob
LNGD	1.0962	0.0000	1.0031	0.0000	6.1649	0.0000
LNGD2	− 0.0300	0.0000	− 0.0143	0.0000	− 0.2918	0.0000
LNECO	0.3273	0.0000	− 0.0234	0.0000	0.3830	0.0009
LNPU	− 0.4356	0.0000	− 0.5049	0.0000	− 1.5044	0.0000
LNPE	0.0181	0.0000	0.0645	0.0000	0.0665	0.0316
LNU	− 0.8994	0.0000	− 0.9617	0.0000	− 3.3421	0.0000
C	− 2.8965	0.0000	− 4.1666	0.0000	− 21.5545	0.0000
*R*^2^	0.9939		0.9772		0.9998	
Adjusted *R*^2^	0.9924		0.9720		0.9989	
S.E. of regression	0.0305		0.0587		0.0112	

**Fig. 6:**
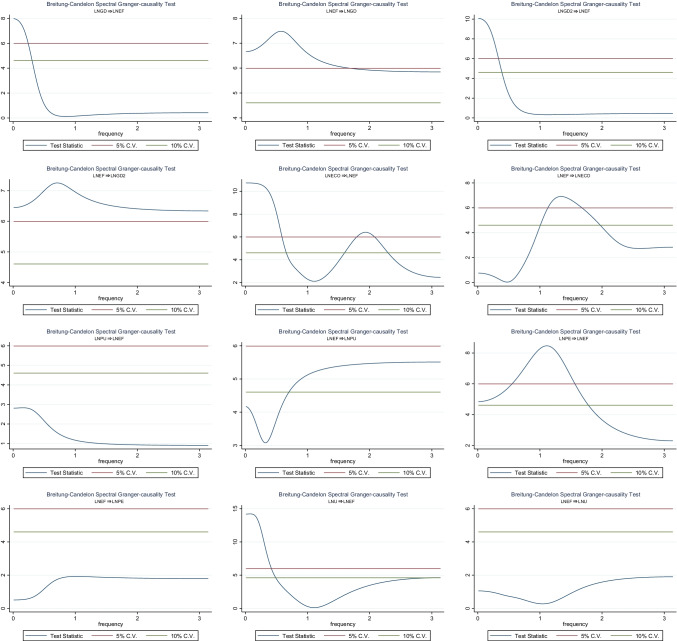
Spectral BC causality between GD, GD2, PU, PE, *U*, and *EF*

This study further uses the FDCt that was motivated by work from Abbasi et al. (2021) to ascertain the linkage between GD, GD2, ECO, PU, PE, and *EF* in China for robustness analysis. As shown in Fig. 6, The occurrence of the bidirectional granger causality relationship between GD, GD2, ECO, PU, PE, *U*, and *EF* were investigated at frequencies 2–3, 1–2, and 0–1. These frequencies show a short, medium, and long-term causal association, and 0–1 and 1–2 is defined as permanent and medium causality, while 2–3 is known as temporary causality effect. A bidirectional granger causality relationship was verified between real income per capita and *EF*, suggesting that the economic development has a long-term ecological degradation drift, and ecological degradation pressure potentially affects economic growth in the medium term, and several factors of *EF* such as industrial pollutants emissions are argued to restrain economic growth in China as well (Rao and Yan, 2020). In addition, the FDCt results validate ECO as an essential driver of environmental degradation at different frequencies, whereas *EF* affects the energy consumption in the medium term. Urbanization in the long- and short-term affects environmental sustainability via different negative and positive effects.

Furthermore, P exports have a favorable influence on the environmental sustainability in the long and short term, suggesting P industry influence mainly embodied in P exports sector, and a one-way granger causality flows from *EF* to PU in the short and medium term, instead of flows from PU to PE are verified from the FDCt results. This result suggests that the acceleration of environmental stress might promote stringent policies of P use management in China`s agricultural sector in the short-and medium term.

## Conclusions, implications, and limitations

### Conclusions

Based on the empirical results of the P-induced EKC framework, we can conclude that the per capita income and EF are associated with the inverted *U*-shaped EKC hypothesis form in 1985–2018 in China. Specifically, it could be shown that the deterioration of the environmental quality decreases after a certain level of per capita income has been reached if key determinants of energy consumption–based carbon emissions and urbanization are incorporated in the analysis of the EF in China’s P industry. With regards to the model selection, our robustness analysis confirmed that the selected ARDL model has excellent fitness and goodness performance, and that this model can serve to provide reliable quantitative results for *EF* analysis of China’s P industry. This study further provides empirical results that contribute to the P-*EF* nexus analysis. Specifically the following conclusions can be drawn from this study:

1. The empirical results indicate a strong long-run cointegration relationship between GD, GD2, PU, PE, and *U* to *EF* at 78.62% speed of adjustment. The indispensable variables of real GDP, energy consumption, urbanization (or trade) are suggested to be incorporate in any future *EF* analysis for different industry sectors to ensure the fitness, goodness, and robustness of the empirical findings.
2. The results verify that a key to the understanding of the identified factors of ecological degradation is rooted in the long- and short-run ECO impact that resulted in an increase of 0.226% and 0.51%. This again implies that short-run effects of ECO on *EF* are confirmed to be significantly higher than long-run effects. ECO also affects the *EF* in the medium term because environmental degradation stress might promote stricter policies to control carbon emissions in China.
3. Our results validate the EF in China’s P industry mostly embodied in the P export sector instead of P use in the long run, and P exports are identified to have a 0.04% long-run effect on *EF*. The scale effect mostly depends on the characteristics of the energy as well as the low- and medium-quality PR resources used in P processing. The decreasing ore grades might even have neutralized the effort of improving PU efficiency in the agricultural sector in recent years in China, as well as potential developments of cleaner P production technologies.
4. The nexus of PU and *EF* is mainly derived from two aspects: PU has a lagged stimulation effect on environmental degradation in the short run. Moreover, in the medium and short term, *EF* has a significant impact on PU, suggesting ecological pressure on PU as a result of the application of stricter policies and regulations used to control PU and to improve the overall efficiency.
5. The other important factor is urbanization, and our results could confirm that different stages of urbanization have different impacts on environmental degradation. Although it was re-confirmed that the *EF* decreases with rising urbanization in the long run, urbanization has a significant positive effect on *EF* in the short run.

### Implications: towards greener and cleaner P exports from China

Although economic growth significantly drives higher-grade natural resources (energy, mineral resources, water) depletion in China, it is obvious that sustainable economic development relies on integrating sustainable natural resources utilization and policies that reduce the effect of negative externality on the environmental sustainability. More importantly, opinion leaders from academic and governmental organizations can put more emphasis on changing more than just carbon emissions resulting from fossil fuel energy usage, but also by considering other ecological degradation factors from different industry sectors, and promoting ecological sustainability initiatives from the experience of the industrial chain to boost the outcomes of achieving environmental benefits among sectors (Xia et al. 2022).

In this context, the present study provides important implications highlighting the interdependence between the output of China’s P industry and environmental sustainability. It is suggested that:

Firstly, more P exports should be allocated to P companies that are committed to greener and cleaner production processes under China’s current P export license management since 2019. More investment in clean energy deployment for P production is encouraged, since fossil energy use is directly related to GHG emissions and expanding clean energy usage is the best alternative solution to achieving economic, social, and environmental sustainability (Ulucak and Lin 2017). In practice, policy makers can formulate policies that encourage the transition from expanding P production capacity to other priorities, such as, improving energy use efficiency, utilizing advanced technologies to increase cleaner production and environmental pollution prevention measures, and implementing larger scale P recycling. Possible policy-level interventions such as subsidies, interest on loans, and export allocation can be considered to help particularly small- and medium-sized P companies in China.

Secondly, considering the pioneering role of China in the global P export market, China can take responsibility and initiatives for deploying sustainable P exports and help trade partners in strengthening their policies to import and promote the use of greener and cleaner P fertilizer. Here, Chinese companies can act and initiate collaborations with other major exporters to jointly tackle the global challenges of cleaner P fertilizer production.

Thirdly, as this is a truly global challenge, we encourage not only international cooperation but also the creation of international standards in P production. These initiatives can be beneficial to create economic benefits and balance the negative environmental externalities associated with P production and P utilization. In this context, we strongly encourage P producing companies to provide P footprint calculations to present their P flows, and thus commit to their environmental responsibility.

Finally, we strongly believe that ***Multidisciplinary, multidimensional and practical solutions*** are key to the transition of China’s P industry. In this context, greater awareness of the debate of the *EF*-P nexus can further enable citizens to better observe economic, social, and industry sectors potentially negatively influence on ecological resources, and then pursuit sustainable environmental practices and regulations (Langnel and Amegavi 2020).

### Limitation

We recommend that future studies investigate and further discuss the understanding of the EKC hypothesis by incorporating different variables, industry sectors in cases of different countries and regions by using different models. Although we broadened the empirical investigation by exploring the analysis of *EF* in China’s P industry, the findings are restricted by data availability and uncertainty. In this study, we could only consider the sampling period from 1985–2018. It is widely known that cointegration models depend on the longitude of available time series datasets. If we include more recent annual data (2019–2022), we can infer that the results might show some difference to the presented findings. Furthermore, although *EF* is widely used and accepted as an indicator of environmental degradation, we call upon organizations and academics to design and develop even more comprehensive and systematic P footprint models that can result in an even better understanding of the underlying processes.

## Data Availability

All data used is openly accessible.

## Footnotes

1 https://www.footprintnetwork.org/content/uploads/2020/08/Footprint-Limitations-and-Criticism.pdf

2 http://www.lomonland.com/index.php?case=archive&act=show&aid=296

3
https://www.weforum.org/agenda/2020/11/china-bans-fossil-fuel-vehicles-electric/.
